# Personal trauma history and secondary traumatic stress in mental health professionals: A systematic review

**DOI:** 10.1111/jpm.13082

**Published:** 2024-07-07

**Authors:** Anita Henderson, Tom Jewell, Xia Huang, Alan Simpson

**Affiliations:** ^1^ Florence Nightingale Faculty of Nursing, Midwifery & Palliative Care King's College London London UK; ^2^ Children & Adolescent Mental Health Central North West London NHS London UK; ^3^ Florence Nightingale Faculty of Nursing, Midwifery and Palliative Care, King's College London, David Goldberg Building London UK; ^4^ Great Ormond Street Hospital NHS Foundation Trust London UK; ^5^ Mental Health Centre, West China Hospital Sichuan University, West China School of Nursing, Sichuan University Chengdu China; ^6^ Care in Long Term Conditions Research Division Florence Nightingale Faculty of Nursing Midwifery & Palliative Care, King's College London London UK; ^7^ Health Service and Population Research Institute of Psychiatry, Psychology, Neuroscience King's College London London UK

**Keywords:** compassion fatigue, mental health professional, personal trauma history, secondary traumatic stress, vicarious trauma

## Abstract

**Introduction:**

Caring for those who have been traumatized can place mental health professionals at risk of secondary traumatic stress, particularly in those with their own experience of personal trauma.

**Aim:**

To identify the prevalence of personal trauma history and secondary traumatic stress in mental health professionals and whether there is an association between these two variables in mental health professionals.

**Method:**

We preregistered the review with PROSPERO (CRD42022322939) and followed PRISMA guidelines. Medline, Embase, PsycINFO, Web of Science and CINHAL were searched up until 17th August 2023. Articles were included if they assessed both personal trauma history and secondary traumatic stress in mental health professionals. Data on the prevalence and association between these variables were extracted. Quality assessment of included studies was conducted using an adapted form of the Newcastle‐Ottawa scale.

**Results:**

A total of 23 studies were included. Prevalence of personal trauma history ranged from 19%–81%, secondary traumatic stress ranged from 19% to 70%. Eighteen studies reported on the association between personal trauma history and secondary traumatic stress, with 14 out of 18 studies finding a statistically significant positive relationship between these variables. The majority of studies were of fair methodological quality.

**Discussion:**

Mental health professionals with a personal history of trauma are at heightened risk of suffering from secondary traumatic stress.

**Implications for Practice:**

Targeted support should be provided to professionals to prevent and/or address secondary traumatic stress in the workforce.

**Accessible Summary**

**What Is Known on the Subject**

- Mental health professionals have typically experienced more traumatic events in their own lives, compared with the general population.
- Mental health professionals work with patients who sometimes share their history and experience of traumatic events.
- Listening to these firsthand accounts of trauma can place the mental health professional at risk of experiencing secondary traumatic stress.
- Secondary traumatic stress refers to symptoms of post‐traumatic stress that are caused by indirect exposure to trauma.

**What the Paper Adds to Existing Knowledge**

- Personal trauma history and secondary traumatic stress are common in mental health professionals.
- Mental health professionals are at higher risk of developing secondary traumatic stress when they have their own experiences of trauma.

**What Are the Implications for Practice**

- Identifying those who are most at risk of developing secondary traumatic stress has implications for education and health care settings.
- Embedding teaching about the possible psychological impacts of secondary traumatic stress for mental health professionals with their own experiences of trauma could lead to improved well‐being of the practitioner and support services to retain skilled staff.
- In clinical services, those at risk of developing secondary traumatic stress in practice should receive targeted help and support, such as specialized supervision and debriefs.

## 1 INTRODUCTION

Mental health professionals' often work with patients who have faced some form of trauma. These patients will either be witness to, or victims of, child abuse, sexual violence, serious injury or threats of death (Ogińska‐Bulik et al., 2021). It has been found that when the patients share their traumatic story with a mental health professional, the empathy the clinician feels towards those who are suffering makes them more vulnerable to experiencing their patient's pain (Beck & T., 2011) and can place them at risk of developing symptoms of post‐traumatic stress disorder (Jacobs, 2017).

For clarity, below we briefly review different terms and how they have been operationalized in research.

### 1.1 Defining secondary traumatic stress and related terms

Over the years trauma symptoms have been described using a number of terms including secondary traumatic stress, vicarious trauma (i.e. cognitive symptoms associated with indirect exposure) and compassion fatigue (i.e. trauma and burnout symptoms) (Greninacher et al., 2019). Burnout (World Health Organisation, 2019) is unrelated to post traumatic stress symptoms but represents a different concept which has both overlap and differences with the other terms. We define these constructs in the following ways:

Post Traumatic Stress Disorder (PTSD) is a mental health diagnosis included in the Diagnostic and Statistical Manual of Mental Disorders (DSM‐5) (American Psychiatric Association, 2013). Criterion A of the diagnostic criteria specify that an individual must have been exposed to a stressor (e.g. death, threatened death, actual or threatened sexual violence) in one of the following ways: (A1) through direct exposure; (A2) witnessing the trauma; (A3) learning that a relative or close friend was exposed to a trauma; (A4) or through ‘experiencing repeated or extreme exposure to averse details of the traumatic event(s)’ (American Psychiatric Association, 2013). The DSM‐5 gives two examples of this A4 criterion: first responders collecting human remains, or police officers exposed to details of child abuse. Whilst examples of mental health professionals meeting criterion A4 are missing, it is theoretically possible for such professionals to meet such criteria. However, as explained below, secondary traumatic stress may be considered a preferable term to differentiate between PTSD and the trauma reactions most likely to be experienced through indirect exposure in mental health professionals (Penix et al., 2020).

#### 1.1.1 Secondary traumatic stress

Secondary traumatic stress is an acute reaction that occurs when professionals become psychologically overwhelmed in their desire to support others (Orrù et al., 2021). Symptoms experienced by the professional often mirrors those of their clients who are suffering from PTSD (Ogińska‐Bulik et al., 2021), but secondary traumatic stress can be differential from PTSD as a subclinical symptom picture, conceptualized as an index of stress rather than a clinical disorder (Penix et al., 2019). To measure for secondary traumatic stress the Secondary Traumatic Stress Scale (STSS) (Bride et al., 2004) is often used. This is a self‐report questionnaire consisting of 17 Likert questions based on secondary post‐traumatic stress symptoms, such as avoidance, intrusion and arousal (Jacobs et al., 2019).

#### 1.1.2 Vicarious trauma

Vicarious trauma refers to mental health professionals' cognitive schema becoming altered when working with patients who have been traumatized and will view new experiences with suspicion and a sense of cynicism (Pearlman & Mac Ian, 1995). Vicarious trauma differs from secondary traumatic stress in that secondary traumatic stress is acute and can occur from a single exposure while vicarious trauma is accumulative (Branson, 2019). While studies on the impact of secondary traumatic stress on mental health professionals' have not used the term ‘cognitive schema’, they report that the clinicians' views of the world are altered (Simon et al., 2006) and they struggle with their ability to continue to care for the patient and express warmth, empathy and understanding (Hoffman, 2009). Vicarious trauma is typically measured using the Trauma Symptom Inventory (TSI) Belief Scale (Jenkins & Baird, 2002) which assess for disruptions in areas for self and others.

#### 1.1.3 Compassion fatigue

Compassion fatigue occurs following prolonged exposure to client's traumatic material where the therapist will often re‐experience their client's traumatic event (Robino, 2019). It is a combination of secondary traumatic stress and burnout, with professionals feeling both mentally and physically exhausted and struggling to cope with everyday life (Figley, 1999). Compassion fatigue is often measured using the Compassion Fatigue Self‐Test (Stamm & Figley, 1996). While the compassion fatigue self‐test is still used in studies it has developed into The Professional Quality of Life Scale which consists of three subscales compassion fatigue, burnout and compassion satisfaction (Stamm, 2010).

#### 1.1.4 Burnout

Burnout is defined as an occupational phenomenon resulting from chronic stress experienced in the workplace (Edú‐Valsania et al., 2022). The cause of burnout differs from secondary traumatic stress and vicarious trauma and is related to organizational pressures placed on staff‐ such as an increased workload and staff shortages, rather than working with traumatized patients. However, the symptoms clinicians experience because of burnout are similar to those who experience secondary traumatic stress, which include feelings of fatigue and detachment (Kanno & Giddings, 2017).

**FIGURE 1:**
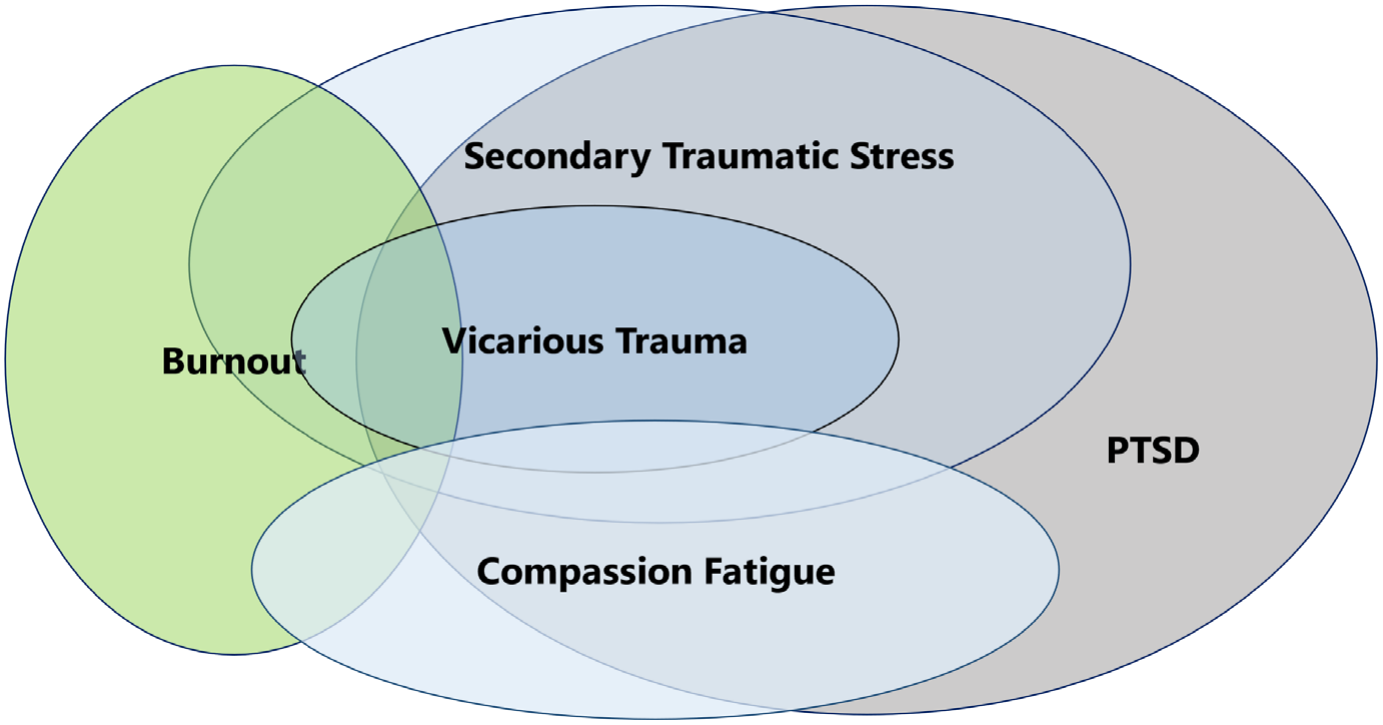
Conceptual diagram illustrating overlaps between trauma concepts and burnout.

These terms have substantial degrees of overlap, and may present with similar symptoms such as fatigue, cynicism, irritability and feelings of hopelessness. Secondary traumatic stress, vicarious trauma and compassion fatigue are all defined as developing in response to contact with traumatized patients, whereas burnout results from organizational pressures. PTSD symptoms can be present across secondary traumatic stress, vicarious trauma, compassion fatigue and burnout, however whereas PTSD is a mental health disorder, the other listed concepts are understood as indices of stress, typically not meeting full criteria for PTSD Figure 1 presents our construction of the overlaps and distinctions between concepts in diagrammatic form.

### 1.2 Personal trauma histories in mental health professionals

Those professionals working in mental health are attracted to work within this area mainly out of a desire to help and support others (McKenzie et al., 2020). The knowledge and expertise required to work in this field is not just learnt from their education and training but is often drawn from their own experiences (Jenkins et al., 2011). It has long been acknowledged within healthcare that those attracted to working in mental health often suffer from their own traumatic life events (Somoray et al., 2017). Chaverri et al. (2018), study of mental health professionals found 109 of the 153 (71.2%) participants had experienced some form of personal trauma.

Many studies have found that those mental health professionals who have experienced a traumatic event in their own life are more likely to experience secondary traumatic stress (Zerach & Ben‐Itzchak Shalev, 2015). When attempting to identify the risk factors for secondary traumatic stress in mental health professionals, numerous studies have found that having a personal history of trauma can increase the risk of a mental health professional experiencing secondary traumatic stress (Hensel et al., 2015); although, other research has found that there is no significant link between secondary traumatic stress and a clinician's own trauma history (Creamer & Liddle, 2005).

Studies vary on how they determine whether a mental health professional has their own history of personal trauma with the majority asking the participant to answer either yes or no to whether they have experienced trauma within their own lives (Brockhouse et al., 2011). Others have formulated their own questions based on symptoms of post‐traumatic stress disorder (Corbett‐Hone & Johnson, 2022). Validated measures such as the Trauma History Questionnaire (Hooper et al., 2011), The Traumatic Attachment Belief Scale (TABS) (Pearlman, 2003), The Life Events Checklist −5 (Gray et al., 2004) and The Impact Event Scale‐revised (Weiss & Marmar, 1997) have also been used.

Previous research has however varied in the use of terms for secondary traumatic stress, measures used and population studied. To ensure a robust and thorough review of the literature the inclusion criteria will include all concepts of secondary traumatic stress and validated measures will be included, along with all mental health professionals.

It is possible that a personal trauma history may make a mental health professional more vulnerable to suffering from secondary traumatic stress as has been found among substance misuse workers (Cosden et al., 2016), in rape crisis staff (Dworkin et al., 2016) and those in the field of medical trauma care (Ogińska‐Bulik et al., 2021). While these are challenging and complex fields of health care, mental health professionals also support some of the most vulnerable patient groups. These patients will often share their history and experience of traumatic events with the clinician. Busy and over‐stretched working environments will often mean there is no direct access to supervision (Rothwell et al., 2021) and the professionals' codes of confidentiality mean they are unable to share with family and friends for support (Nursing & Midwifery Council, 2018).

If we consider then that those who are attracted to work in the field of mental health care often share similar traumatic experiences to their patients and that there is a likelihood, they are at a heightened risk of suffering from secondary traumatic stress it can be argued that the impact on their health is a concern and may have an impact on retention.

To our knowledge there has been one previous systematic reviews of the literature on secondary traumatic stress and personal trauma history in mental health professionals. This is Leung et al. (2022), review on a personal history of trauma and experience of secondary traumatic stress, vicarious trauma and burnout in mental health workers. Leung et al. (2022) study identified 26 quantitative studies of personal trauma history and secondary traumatic stress in mental health professionals, of which 17 reported a positive association. A table has been placed in the supporting documents to show the differences between this review and the student researchers. Fundamentally however Leung et al. (2022) has included burnout as variable and states that burnout is caused by exposure to a client's traumatic experiences when burnout has been clearly defined in the literature as being caused being caused by organizational pressures such as the impact of short staffing resulting in high caseloads and poor retention of staffing (Rayner et al., 2020). Leung et al. (2022) also acknowledges compassion fatigue as being closely linked to secondary trauma and include it as a search term but do not report on compassion fatigue findings. In addition, the definition of mental health professionals was broad, and included volunteers, advocates, child protection workers and those who work in protective services. In particular, the review included nine studies which focussed or included non‐clinical social workers who were not working in a mental health setting. Only a single reviewer was used unless there were queries regarding the inclusion criteria. There were also limitations placed on the included number of studies being captured with the first 1000 being collected and the range was between years 2000 to June 2021. The review did stipulate if the studies included needed to use a valid tool to assess for vicarious trauma or secondary traumatic stress and did not report the prevalence of personal trauma history and secondary traumatic stress, which is crucial data for both clinical practice settings and researchers. Finally, the quality assessment of studies was not reported at the item‐level, only as a summary score, meaning that there was a lack of detail about the strengths and limitations of individual studies.

## 2 AIMS

The review aimed to: (1) identify the prevalence of personal trauma and secondary traumatic stress in mental health professionals and: (2) identify whether there is an association between personal trauma history and secondary trauma in mental health professionals.

## 3 METHODS

We undertook a systematic review following PRISMA guidelines (Page et al., 2021), and pre‐registered our protocol in PROSPERO.

### 3.1 Search strategy and selection criteria

**FIGURE 2:**
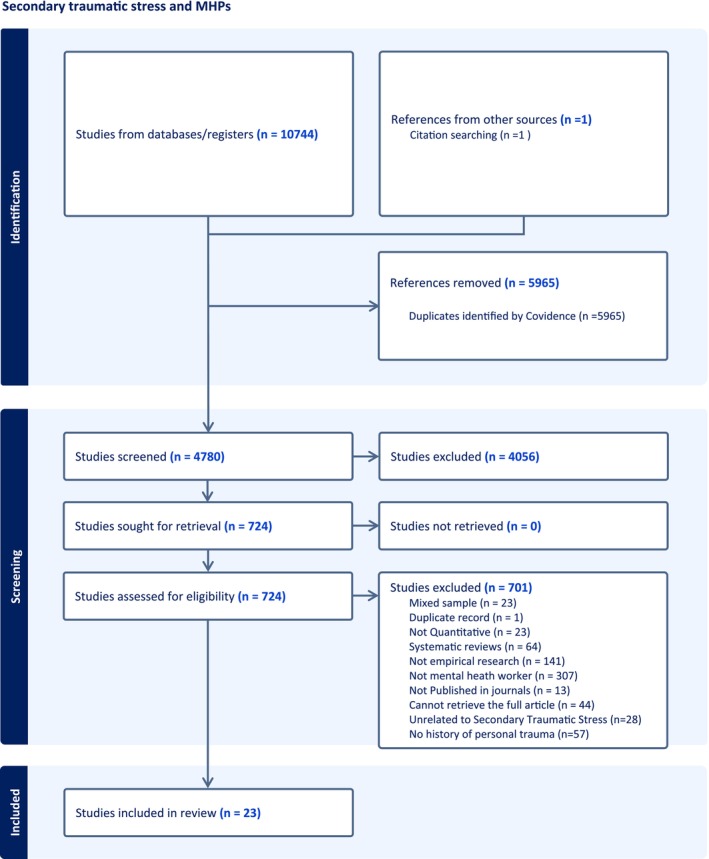
PRISMA flowchart.

The systematic review was undertaken following a pre‐registered protocol on PROSPERO (CDR42022322939). With the support of an independent librarian the Population, Exposure and Outcome (PEO) framework was used to define concepts and terms (Bettany‐Saltikov & McSherry, 2016) (Table 1). Medline, EMBASE, PsyINFO, Web of Science and CINHAL were searched using the following keywords in combination (seconda*trauma OR secondary traumatic stress OR compassion fatigue OR vicarious trauma*). Rather than mental health professional and personal trauma being used as keywords these were screened for using the title and abstract. Following the findings of a scoping review undertaken by one of the authors (AH), an intentionally broad search strategy was used because of the nonspecific use of outcome measures for measuring personal trauma in the literature (Hensel et al., 2015) and the wide variety of professional titles for Mental Health Professionals (Table 1). Results were reported adhering to PRISMA guidelines (Figure 2). Searchers were conducted on the 1st April 2022, and repeated across all databases on the 17th August, 2023. Studies were imported into Covidence, and deduplicated prior to articles being independently screened by two authors (AH) and (XH) in two stages. Firstly, using titles and abstracts and then full text records. Non‐consensus was resolved by a third reviewer (TJ). Finally, we searched the reference list of studies assessed during the full‐text stage to identify additional studies. In addition, Google Scholar was searched using the same keywords and published review articles on Secondary Traumatic Stress were surveyed to identify any appropriate articles not found in the database search.

**TABLE 1:** Population, exposure and outcome with inclusion & exclusion rationale.

PEO	Inclusion	Exclusion	Rationale
Population: Mental health professionals	Mental Health Nurses Clinical Social workers Clinical and counselling Psychologists Psychotherapists Psychiatrists Counsellors	Non‐clinical social workers. Those working within protective and victim services, child welfare, volunteers, providers and interviewers.	Mental Health Professionals was not used as a search term due to the wide variation of the term used in practice. This was screened using a robust inclusion and exclusion criteria and reviewers' expertise from working in this field.
Exposure: Personal trauma	‘An event, or series of events, or set of circumstances that is experienced by an individual as physically or emotionally harmful or life threatening and that has lasting adverse effects on the individual's functioning and mental, physical, social, emotional, or spiritual well‐being’ (Substance Abuse and Mental Health Services Administration, p.7, 2014).		An intentionally broad search was used for Mental health professionals because of the nonspecific use of outcome measures across the various terms used in the literature (Hensel et al., 2015)
Outcome: Secondary traumatic stress	Secondary traumatic stress		Secondary traumatic stress is the behavioural and emotional consequences of exposure to traumatic events experienced by significant others (Figley, 1995). It is characterized by Post Traumatic Stress Disorder (PTSD) symptoms and been recognized in the Diagnostic Statistical of Mental Disorders, 5th Edition (DSM‐5) (American Psychiatric Association., 2013).
	Vicarious trauma		Wilson and Lindy (1994) describe vicarious trauma as a form of PTSD with Herman and Harvey (1997) stating vicarious trauma is the traumatic countertransference form with the therapist experiences the same terror, rage and anguish as the patient.
	Compassion fatigue		Compassion fatigue closely relates to PTSD symptoms (Hensel et al., 2015). While it commonly conceptualized by having two dimensions Secondary Traumatic stress and Burnout (Rauvola et al., 2019), it is often used interchangeably with Secondary Traumatic stress (Greninacher et al., 2019), with the latter deemed a more user‐friendly term (Figley, 1995).
		Burnout	Is not specific to exposure to traumatic material and can affect individuals in any professional role as it develops in the setting of prolonged exposure to stressful demands at work (Cieslak et al., 2013)

Inclusion/exclusion criteria (maybe seen in Table 1).

*Studies were included if they*

- Assessed either personal trauma history OR secondary trauma, secondary traumatic stress, vicarious trauma, or compassion fatigue (Table 1).
- Reported on one or both of the following:

- The correlation between personal trauma history and secondary trauma/secondary traumatic stress/vicarious trauma/compassion fatigue.
  And/or
- The prevalence of either personal trauma history and/or secondary trauma/secondary traumatic stress/vicarious trauma/compassion fatigue (Table 1).

- The sample was comprised of mental health professionals. These professionals included mental health nurses, psychiatrists, social workers, psychologists, occupational therapists and counsellors who have experienced their own trauma.
- The sample also included student mental health professionals of the professional background mentioned above if they had placements in a mental health setting and who have experienced their own trauma.
- Searches were limited to empirical articles published in English.
- Only valid measures producing quantitative data to assess the prevalence of secondary traumatic stress, secondary trauma, vicarious trauma and compassion fatigue were included.
*Studies were excluded if*;
- Reported on burnout only (Table 1).
- We excluded qualitative studies, books, book chapters, personal blogs, commentaries, conference abstracts and thesis.

### 3.2 Data extraction and analysis

Information was extracted independently using a standardized pre‐piloted form for all studies by the two reviewers (AH, XH). Data extracted included author, date of publication, title of the article, country, study focus, study population, design, response rate, sample size, measure used and prevalence. The correlation® between personal trauma and secondary trauma was extracted where possible. Calculation of reliability was dependent on percentage of consensus between investigators when searching and screening articles. Any variance was discussed by the investigators (AH & XH) but, if unable to reach an agreement reviewer (TJ) was asked to make the final decision.

Study authors were contacted and asked to provide the following information where this was missing: Pearson's correlation and the p value; and the percentage of participants who report (a) history of personal trauma and (b) secondary traumatic stress.

### 3.3 Risk of bias (quality) assessment

Two review authors (AH, XH) independently assessed the risk of bias in included studies using the Newcastle–Ottawa Scale (Wells et al., 2013) for cross sectional, case control and cohort studies, measuring methodological quality and risk of biases for quantitative studies through the awarding of points by focusing on the representativeness and selection of the sample, comparability of the participants and assessment of the outcome (95% agreement between reviewers). Any disagreements were resolved by discussion or with involvement of a third author (TJ) were necessary. The Newcastle‐Ottawa was adapted for this study and can be found in Data S1. Here, studies would score 1 if the tool to measure for personal trauma history (Ascertainment of exposure) was valid and/or well described. Then, if the assessment tool used to assess for secondary traumatic stress (Ascertainment of outcome) was the Professional Quality of Life/STSS/The Impact of Event Scale/The Traumatic Attachment Belief S‐5/Secondary Traumatic Event Scale/Post‐traumatic disorder Check list/The TSI Belief Scale (Revised) a score of 2 was given. If a different measure was used a score of 1 was given and then 0 for non‐validated measures. Scoring method is available in Data S1.

### 3.4 Data synthesis

Due to the heterogeneity of the studies included in the review, a meta‐analysis was not possible. Instead, a narrative synthesis was completed as recommended by Popay et al. (2006). Findings were organized according to the key research questions: the association between personal trauma history and secondary traumatic stress; the prevalence of personal trauma history in mental health professionals; the prevalence of secondary traumatic stress in mental health professionals. The study focus, study design and quality were also reported on within the data synthesis.

## 4 RESULTS

### 4.1 Study selection

Database searches resulted in 4779 original articles, leaving 678 for full text review following title and abstract screening. Twenty‐two studies met the inclusion criteria, with the addition of one further study found by reviewing the reference list. The final data set consisted of 23 peer reviewed articles. A PRISMA diagram is shown in Figure 2 and a summary of the main characteristics of the studies in Table 2.

**TABLE 2:** Study characteristics.

First author year	Country	Sample	Aim	Design and measures	Sampling strategy & characteristics	Key findings	Quality appraisal
Adams & Riggs (2008)	USA	Trainee therapists (*n* = 129) (Female 108). State Universities in Texas.	Explore VT among therapists in relation to history of trauma, experience level, trauma‐specific training & defence style.	Cross‐sectional survey. Measure of STS: TSI Measure of PTH: Own questions Descriptive statistics	Purposive sampling Trauma clients	STS: 31% PTH: 38.7% Positive association between PTH & STS.	5
Buchanan et al.(2006)	Canada	Trauma therapists (*n* = 280) (Female 235). Community agencies (56%), Private (24%), MH agencies (16%), hospital (16%), other (29%).	To collect data looking at work setting data, client type variables, personal history of trauma, current levels of traumatic stress symptoms and STS reported.	Cross sectional survey Measure of STS: CFST, IES‐R & self‐reported ratings on frequency of experiencing STS (7 questions). Measure of PTH: IES‐R (2 questions)	Sampling approach not reported. Trauma clients	STS: 60% PTH: 61% emotional or psychological abuse, child sexual abuse 32%, child physical abuse 25%, Accidental Disaster 43%, Domestic abuse 30%, Rape during adulthood 14%. Association between PTH & STS not documented.	4
Chaverri et al. (2018)	USA	Mental Health Care Provider (*n* = 153) (Female 103) Does not state.	Investigate the relationship between MHP's personal history of trauma, happiness, and the effects of seeing clients with similar trauma.	Cross sectional Measure of STS: PCL Civilian & Military versions. Measure of PTH: Own Questions	Sampling approach not reported. Trauma caseload (56.2%)	STS: PCL‐M score M (SD) 29.77 (16.03), range from 17 to 71 STS: PCL‐C score M (SD) 25.16 (7.60), range from 17 to 58 PT: 71.2% Association between PTH & STS not documented.	5
Cieslak et al. (2013)	USA	Clinical Psychologists, Counsellors, Social Workers (*n* = 224) (Female 149) On post providers (57%), Off post providers (43%).	Assess the prevalence of STS.	Cross sectional Measure of STS: STSS Measure of PTH: STES developed for this study.	Sampling approach not reported. Military patients.	STS: 19.2% PTH: 3.24 mean SD 1.84, range 0–10 Positive association between PTH & STS B = .17, *p* = .004.	5
Corbett‐Hone & Johnson(2022)	USA	Mental Health Providers (*n* = 89) (Female 85) National: Specific areas not stated.	Examine the prevalence and predictors of STS, Vicarious resilience, BO & CS.	Cross sectional Measure of STS: STSS Measure of PTH: Own questions	Convenience sampling Human trafficking survivors.	STS: 30.3% PTH: 83.1% Positive association between PTH & STS.	5
Creamer & Liddle (2005)	USA & Canada	Mental Health Professionals (*n* = 80) (Females 50) National: not stated.	Relationship between STS symptoms and therapists characteristics and assignment variables.	Cross sectional Measure of STS: IES Measure of PTH: LEC	Sampling approach not reported. Affected by the terrorist attacks of September 2011.	STS: not reported. PTH: not reported. Association between PTH & STS not significant *r* = .17	5
Devilly et al. (2009)	Australia	Mental Health Professionals (*n* = 152) (Gender not stated) Setting not stated.	To perform an assessment of STS & VT and workplace BO.	Cross sectional Measure of STS: STSS & TSI‐BSL Measure of PTH: Own questions	Randomized sampling Trauma client caseload M (SD) 37.54 (32.31) max 100.	STS: not reported. PTH: M (SD) 2.55 (3.18) range 0–22 No association between PTH & STS (*r* = .08).	6
Diehm et al. (2019)	Australia	Psychologists (*n* = 78) (Female 65). Clinical Independent practice settings (52%)	Explore the relationship between personal history of trauma, years of professional experience, level of exposure, age and the development of STS and to examine whether social support acts as a moderating factor	Cross sectional Measure of STS: STSS Measure of PTH: Own questions	Sampling approach not reported. Trauma client caseload M (SD) 25.28 (18.41)	STS: M (SD) 2.02 (0.80) PTH: M (SD) 4.40 SD 2.49 PTH positively associated with STS *r* = .37 (*p* < .001).	3
Dunkley & Whelan (2006)	Australia	Telephone Counsellors (*n* = 62) (Female 55) National	Investigate the influence of coping style, supervision and personal trauma history on VT.	Cross sectional Measure of STS: TABS & IES‐R Measure of PT: TABS	Sampling approach not reported. Trauma phone calls	STS: Tabs 98%, M (SD) 45.28 (8.18) and IES‐R 94%, M (SD) 9.21 (10.36) PTH: 37.1% PTH positively associated with STS using IES‐R total score (*r* = .28) (*p* < .05)	5
Iyamuremye & Brysiewicz (2015)	Rwanda	Mental Health Workers (*n* = 180) (Females 121) Setting not stated	To develop a comprehensive model to manage the effects of STS	Cross sectional measure of STS & PTH: TABS	Convenience sampling Trauma clients	STS & PTH: M (SD) 77.0 (1.2) PTH: 73.8% Experienced genocide, 10% accidental disaster, 7.7% emotional & psychological abuse, 7.2% natural disaster, 2.2% physical abuse as a child. Association between PTH & STS not reported.	2
Killian (2008)	USA	Counselling Services (*n* = 104) (Gender not stated) Not stated	Focus on therapists' stress and coping factors related to resilience and BO.	Cross sectional Measure of STS: PRO‐QOL 3. Measure of PTH: THQ	Sampling approach not reported. Child sexual abuse survivors & Adult survivors of domestic violence.	STS: not reported. PTH: not reported. PTH positively associated with STS (*r* = .234) (*p* < .01).	4
La Mott & Martin (2019)	USA	Mental Health Providers (*n* = 371) (Female 349) Not stated	Examine the moderating effects of self‐care on various compassion outcomes.	Cross sectional Measure of STS: ProQOL‐5 Measure of PTH:ACE	Sampling approach not reported. Childhood trauma victims and their families.	STS: M (SD) = 20.93 (5.61) PTH: M (SD) 2.71 (2.23) PTH positively associated between STS: M (SD)= 21.20 (5.77) t(369) = −2.02, *p* = .044	5
Linley & Joseph (2007)	UK	Therapists (*n* = 156) (Female 122). Individual practice (41%), Clinic (4.5%), hospital (4.5%), combined (3%).	Explore both the positive aspects (personal growth, CS) and negative aspects (CF& BO) of therapists' well‐being.	Cross sectional Measure of STS: ProQOL. Measure of PTH: Own question yes/no	Randomisation Does not state	STS: M (SD) = 10.27 (4.80) PTH: not reported No association between PT & STS.	4
MacRitchie & Leibowitz (2010)	South Africa	Trauma Workers (*n* = 64) (Gender not stated). Victim support groups, trauma clinic, lifeline and private (% not stated).	Explore the psychological impact on trauma workers who work with ‘victims’ of violent crimes, specifically focussing on the level of exposure to traumatic material, level of empathy; level of perceived social support and their relation to STS.	Cross sectional Measure of STS: TSI‐BLS & CFS Measure of PTH: Own questions	Non‐probability Trauma caseload	STS not reported. PTH: 50% Positive association between PTH & STS.	4
Makadia et al. (2017)	UK	Clinical Psychology Trainees (*n* = 564) (Female 507). National % not stated.	Investigate the relationship between exposure to trauma work and well‐being (general psychological distress, trauma symptoms & disrupted beliefs).	Cross sectional Measure of STS: STSS, TABS Measure of PTH: TSQ	Sampling approach not reported. Trauma cases 0 (29.8%) 1–2 (42.9%) 3–4 (13.7%) 5–6 (7.1%) 7–8 (3%) 9–10 (1.4%) 11+ (2.1)	STS: STSSM(SD) = 25.60 (7.70)/ Tables M(SD) = 175.9 (37.51)/TSQ M(SD) = 2.99(2.57) PTH: not reported Positive association between STS and PT r = .09	5
Mangoulia et al. (2015)	Greece	Psychiatric Nurses (*n* = 174) (Females 122). Public Health Hospitals (100%)	To investigate the prevalence of STS, CS & BO in psychiatric nurses and their risk factors.	Cross sectional Measure of STS: ProQOL‐5 Measure of PTH: Own questionnaire	Sampling approach not reported. Trauma cases not stipulated participants work in a psychiatric in‐patient setting.	STS: 44.8% PTH: 38.5% Association between PTH & STS not reported	7
McKim & Smith‐Adcock (2014)	International	Trauma Counsellors (*n* = 98) (Female 73). Not stated.	To examine the trauma counsellors' individual characteristics as well as workplace conditions to determine their influence on CF & CS.	Cross sectional Measure of STS: ProQOL(two subscales) Measure of PTH: SLES	Sampling approach not reported. Trauma caseload	STS: M (SD) 11.03 (6.13) PTH: M (SD) 55.09 (33.19) Positive association between PTH & STS.	5
Pearlman & Mac Ian (1995)	International	Trauma Therapists (*n* = 188) (Female 136) Not stated	Examine VT—the deleterious effects of trauma therapy.	Cross sectional Measure of STS: TSI & IES‐R Measure of PTH: Own questions	Sampling approach not reported. Trauma caseload	STS not reported. PTH: 60% Positive association between STS and PTH: M (SD) 190(38) *p* < .05	5
Ray et al. (2013)	Canada	Mental Health Professionals (*n* = 169) (Females 138) Inpatients & Community (% not stated)	Determine the relationship among CS, CF, work life conditions and BO.	Cross sectional Measure of STS: ProQOL (2 subscales) Measure of PTH: Own questions	Convivence sampling Mental health care—trauma cases not stipulated.	STS: M (SD) 11.83 (6.74) PTH: 27.8% No association between PTH & STS	6
Rayner et al. (2020)	Australia	Mental Health Workers (*n* = 190) (Female 177). Not stated	Examine STS and related factors of empathic behaviour and trauma caseload.	Cross sectional Measure of STS: STSS Measure of PTH: Own questions Inferential Statistics	Purposive sampling Trauma caseload	STS: M (SD) 33.93 (10.95) PTH: M (SD) 4.70 (2.72) Positive association between STS & PTH (*r* = .146, *p* = .045)	4
Rossi et al. (2013)	Italy	Mental Health Workers (*n* = 260) (Female 166). Community Mental Health teams (100%).	Assess BO, CF, CS among staff at four community based mental health services.	Cross sectional Measure of STS: ProQOL‐3 Measure of PTH: Own questions	Sampling approach not reported. Mental health in the community –percentage/number of trauma clients not stated	STS: not reported. PTH: One event 24.8%, more than one event 8.9%. Positive association between PTH & STS: One event 10.1 (range 8.6–11.7), more than one 13.5 (range 11.0–16.1) *p* = .031	7
Sodeke‐Gregson et al. (2013)	UK	Therapists (*n* = 253) (Female 182). Specialist trauma services (22.5%), Secondary care (62.5%), other (5%). All NHS.	Assess the prevalence and predictor variables for CS/ BO and STS in a group of UK therapists working with adult trauma clients.	Cross sectional Measure of STS: ProQOL‐5 Measure of PTH: Own questions	Sampling approach not reported. Adult trauma clients.	STS: 70% PTH: 59.3% Association between PTH & STS not reported	6
Somoray et al. (2017)	Australia	Mental Health Workers (*n* = 156) (Female 124). Not stated	Examine the role of personality and workplace belongingness in predicting CS, STS & BO.	Cross sectional Measure of STS: ProQOL‐5 Measure of PTH: Own questions	Sampling approach not reported. Not stated	STS: M (SD) 20.90 (5.07) PTH: 21.2% Positive association between PTH & STS *r* = .24 *p* < .01	5

Abbreviations: ACE, Adverse Childhood Experiences Questionnaire; BO, Burnout; CFS, Compassion Fatigue Self‐test; CS, Compassion Satisfaction; IES, The Impact of Events Scale; IES‐R, The Impact of Event Scale Revised; LEC, Life Events Scale; MH, Mental Health; NHS, National Health Service; PCL, Posttraumatic Stress Disorder Checklist, Military and Civilian version; PTH, Personal Trauma History; PTGI, The Post Traumatic Growth Inventory; Pro‐QOL, The Professional Quality of Life Scale; Pro‐QOL 3, The Professional Quality of Life Scale‐ Version 3; Pro‐QOL 5, Professional Quality of Life Scale‐Version 5; SLES, The Stressful Life Experiences‐Short form; STS, Secondary Traumatic Stress; STSS Secondary Traumatic Stress Scale; STES, Secondary Trauma Exposure Scale; TABS, The Traumatic Attachment Belief Scale; TSI, Trauma Symptom Inventory; TSI‐BSL, The Traumatic Stress Institute Belief‐Scale Revision‐ L; THQ, Trauma History Questionnaire; TSQ Trauma Screening Questionnaire; VT, Vicarious Trauma.

### 4.2 Risk of bias

The adapted Newcastle Ottwa Scale was used to assess the qualities of the studies included in the review. Findings are available in Table 3. In general, studies were of moderate quality. Out of a total score of 8, with high scores representing low risk of bias, five studies scored 6 or above, 17 studies scored between 3 and 5, and 1 study scored between 0 and 2. We found that the majority of the studies sample size were representative of their target sample with the exception of (Diehm et al., 2019; Iyamuremye & Brysiewicz., 2015; Killian, 2008; & Ray et al., 2013). However, the sample size was justified and deemed satisfactory in only four studies (Devilly et al., 2009; Linley & Joseph, 2007; Makadia et al., 2017 & Pearlman & Mac Ian, 1995). The non‐response rate was defined and deemed satisfactory and characteristics of responders in three studies (Corbett‐Hone & Johnson, 2022; Mangoulia et al., 2015; Pearlman & Mac Ian, 1995). The non‐response rate compared in all other than (Buchanan et al., 2006; Corbett‐Hone & Johnson, 2022; Diehm et al., 2019; Iyamuremye & Brysiewicz, 2015; Linley & Joseph, 2007; MacRitchie & Leibowitz, 2010; Makadia et al., 2017). All of the studies used a robust tool to assess secondary traumatic stress, as per inclusion criteria. The outcome per group was reported appropriately in all other than Pearlman and Mac Ian (1995), Iyamuremye and Brysiewicz (2015) and Killian (2008).

**TABLE 3:** Quality assessment.

First author	Date	Study type	Sampling	Sample size	Non‐response	Ascertainment of exposure (PTH)	Assessment of outcome (STS)	Outcome per group reported
Adams	2008	C	1	0	0	1	2	1
Buchanan	2006	C	1	0	0	1	1	1
Chaverri	2018	C	1	0	0	1	2	1
Cieslak	2013	C	1	0	0	1	2	1
Corbett‐Hone	2022	C	1	0	0	0	2	1
Creamer	2005	C	1	0	0	1	2	1
Devilly	2009	C	1	1	0	1	2	1
Diehm	2019	C	0	0	0	0	2	1
Dunkley	2006	C	1	0	0	1	2	1
Iyamuremye	2015	C	0	0	0	0	2	0
Killian	2008	C	0	1	0	1	2	0
La Mott	2019	C	1	0	0	1	2	1
Linley	2007	C	1	0	0	0	2	1
MacRitchie	2010	C	1	0	0	0	2	1
Makadia	2017	C	1	1	0	0	2	1
Mangoulia	2015	C	2	0	1	1	2	1
McKim	2014	C	1	0	0	1	2	1
Pearlman	1995	C	1	0	1	1	2	0
Ray	2013	C	1	1	0	1	2	1
Rayner	2020	C	0	0	0	1	2	1
Rossi	2013	C	2	0	1	1	2	1
Sodeke‐Gregson	2013	C	1	1	0	1	2	1
Somoray	2017	C	1	0	0	1	2	1

*Note*: C, cross‐sectional; +, higher risk of bias; −, lower risk of bias; blank if not applicable.

### 4.3 Characteristics of the studies

All studies were cross‐sectional in design collecting data at one time point. Two studies used mixed methods designs, collecting and analysing both quantitative and qualitative data within the same study, and for which only the quantitative results were used in this review (Iyamuremye & Brysiewicz, 2015; Killian, 2008). Studies were almost all conducted in Western countries, with the most frequent locations being USA *n* = 6, Australia *n* = 5, UK *n* = 3, Canada *n* = 2 (USA & Canada *n* = 1). Two recruited international samples (McKim & Smith‐Adcock, 2014; Pearlman & Mac Ian, 1995) and two studies were conducted in Africa (Iyamuremye & Brysiewicz, 2015; MacRitchie & Leibowitz, 2010).

### 4.4 Participant characteristics

Professional titles of the participants in the studies varied. These were: mental health workers (Iyamuremye & Brysiewicz, 2015; Rayner et al., 2020; Rossi et al., 2013; Somoray et al., 2017), mental health professionals (Creamer & Liddle, 2005; Devilly et al., 2009; Ray et al., 2013), mental care providers (Chaverri et al., 2018), mental health providers (La Mott & Martin, 2019) and clinicians (Killian, 2008), trauma counsellors (McKim & Smith‐Adcock, 2014), trauma therapists (Buchanan et al., 2006; Pearlman & Mac Ian, 1995), therapists (Linley & Joseph, 2007; Sodeke‐Gregson et al., 2013); trauma workers (MacRitchie & Leibowitz, 2010); mental health nurses (Mangoulia et al., 2015), psychologists (Diehm et al., 2019), clinical psychologists and clinical social workers (Cieslak et al., 2013), trainee therapists (Adams & Riggs, 2008) clinical psychology trainees (Makadia et al., 2017) and telephone counsellors (Dunkley & Whelan, 2006). Only three studies did not report on gender (Devilly et al., 2009; Killian, 2008; MacRitchie & Leibowitz, 2010). For all other studies the majority of the participants were female.

### 4.5 Study characteristics

The term secondary traumatic stress was used in 11 studies (Buchanan et al., 2006; Cieslak et al., 2013; Creamer & Liddle, 2005; Diehm et al., 2019; Iyamuremye & Brysiewicz, 2015; La Mott & Martin, 2019; MacRitchie & Leibowitz, 2010; Mangoulia et al., 2015; Rayner et al., 2020; Sodeke‐Gregson et al., 2013; Somoray et al., 2017). The term compassion fatigue was also used (five studies) (Killian, 2008; Linley & Joseph, 2007; McKim & Smith‐Adcock, 2014; Ray et al., 2013; Rossi et al., 2013) as was vicarious trauma (three studies) (Adams & Riggs, 2008; Dunkley & Whelan, 2006; Pearlman & Mac Ian, 1995). One study used post‐traumatic stress disorder (Chaverri et al., 2018) and two separated secondary traumatic stress and vicarious trauma (Devilly et al., 2009; Makadia et al., 2017).

Secondary traumatic stress was typically measured with the Professional‐Quality of Life scale (Pro‐QOL) which identifies symptoms of compassion fatigue, compassion satisfaction and burnout. This was the original (Linely & Joseph., 2007) and updated versions; PRO‐QOL‐3 (Killian, 2008; Rossi et al., 2013) and PRO‐QOL‐5 (La Mott & Martin, 2019; Mangoulia et al., 2015; Sodeke‐Gregson et al., 2013; Somoray et al., 2017), or the Compassion Fatigue subscale of the PRO‐QOL (McKim & Smith‐Adcock, 2014; Ray et al., 2013). Four of the studies used the STSS to capture symptoms of secondary traumatic stress (Cieslak et al., 2013; Corbett‐Hone & Johnson, 2022; Diehm et al., 2019; Rayner et al., 2020). Some studies used single rating scales, such as the TSI (Adams & Riggs, 2008), the Impact of Events Scale (IES) (Creamer & Liddle, 2005) and TABS (Iyamuremye & Brysiewicz, 2015). Others used multiple questionnaires to measure secondary traumatic stress (e.g.Buchanan et al., 2006; Chaverri et al., 2018; Devilly et al., 2009; Dunkley & Whelan, 2006; MacRitchie & Leibowitz, 2010; Makadia et al., 2017; Pearlman & Mac Ian, 1995).

When asking MHP's whether they had experienced trauma in their lives two studies asked the participants to state either yes or no (Linley & Joseph, 2007; Pearlman & Mac Ian, 1995). Some of the studies asked their own original questions to investigate whether the MHP's had a history of trauma. Some used only one question; whether the participant had ever received a formal diagnosis of Post‐Traumatic Stress Disorder (Corbett‐Hone & Johnson, 2022); or experienced direct exposure to violent crimes (MacRitchie & Leibowitz, 2010). Another study asked questions concerning history of trauma and trauma resolution (Diehm et al., 2019), and then personal history of trauma and resolutions of personal trauma (Makadia et al., 2017).

Some of the studies used ratings. In one study, participants rated their personal trauma history from 0 to 10, with 0 representing no trauma and 10 representing extreme personal trauma history (Rayner et al., 2020); another asked about eight lifetime traumatic events‐for example, have you ever been attacked with a weapon?‐and then coded these into categories (none, one event or more than one event) (Rossi et al., 2013).

Some authors asked a number of questions. For instance, Adams & Riggs. (2008) asked questions about whether the participant had personally been involved in a natural disaster, witnessed or been a participant in combat, been a victim of violent crime, a victim of physical, sexual or emotional abuse as a child, an adult victim of sexual assault or rape, been involved in a physically abuse relationship, or witnessed someone being seriously injured or killed. Somoray et al. (2017) asked participants to state whether their history of trauma was personal or work‐related, and to give descriptions of the trauma experienced, as well as the perceived severity of the trauma, defining a traumatic event according to the DSM‐5, 4th edition (American Psychiatric Association., 1994) Ray et al. (2013) asked if they had a history of trauma related to childhood physical abuse/sexual abuse/psychological abuse emotional abuse or neglect.

Mangoulia et al.'s. (2015) precise questions were not listed. They asked participants if they had experienced a traumatic event in which they could have been killed or if they had experienced the death of a loved one. Sodeke‐Gregson et al. (2013) asked their own questions but did not give an example of these.

Cieslak et al. (2013) developed the Secondary Traumatic Exposure Scale for their study. Devilly et al. (2009) adapted the STSS. Buchanan et al. (2006) used two questions from the IES‐Revised (IES‐R). Creamer and Liddle (2005) used the Life Events Scale (LEC); Dunkley and Whelan (2006) the Trauma Scale Inventory (TABS); Killian (2008) Trauma History Questionnaire (THQ); La Mott and Martin (2019) Adverse Childhood Experiences (ACE); McKim and Smith‐Adcock (2014) the Stressful Life Experiences Short Form (SLES); and Chaverri et al. (2018) Post‐traumatic Stress Disorder Checklist Civilian and Military versions (PCL).

### 4.6 Association between personal trauma history and secondary traumatic stress

The majority of studies (14) found a positive association between secondary traumatic stress and a mental health professionals experience of their own personal trauma history (Adams & Riggs, 2008; Cieslak et al., 2013; Corbett‐Hone & Johnson, 2022; Diehm et al., 2019; Dunkley & Whelan, 2006; Killian, 2008; La Mott & Martin, 2019; MacRitchie & Leibowitz, 2010; Makadia et al., 2017; McKim & Smith‐Adcock, 2014; Pearlman & Mac Ian, 1995; Rayner et al., 2020; Rossi et al., 2013; Somoray et al., 2017). Four studies found that there was no association between a mental health professionals secondary trauma history and personal trauma history (Creamer & Liddle, 2005; Devilly et al., 2009; Linley & Joseph, 2007; Ray et al., 2013). Five studies did not report on this at all (Buchanan et al., 2006; Chaverri et al., 2018; Iyamuremye & Brysiewicz, 2015; Mangoulia et al., 2015; Sodeke‐Gregson et al., 2013). While in a few studies the effect size for the correlation was reported to be small in magnitude ranging from *r* = .146, *p* = .045 (Rayner et al., 2020) to *r* = .37 & *p* < .001 (Diehm et al., 2019).

### 4.7 Secondary traumatic stress

Three quarter of the studies reported that significant levels of secondary traumatic stress were experienced by mental health professionals (Chaverri et al., 2018; Diehm et al., 2019; Iyamuremye & Brysiewicz, 2015; La Mott & Martin, 2019; Linley & Joseph, 2007; Makadia et al., 2017; Mangoulia et al., 2015; McKim & Smith‐Adcock, 2014; Ray et al., 2013; Rayner et al., 2020; Somoray et al., 2017). Those who reported their results in percentages Buchanan et al., 2006 (60%); Dunkley & Whelan, 2006 (37.1%); Adams & Riggs, 2008 (31%); Cieslak et al., 2013 (19.2%); Sodeke‐Gregson et al., 2013 (70%); and Corbett‐Hone & Johnson, 2022 (30.3%).

### 4.8 Personal trauma history

For 19 out of the 23 studies that reported on the prevalence of personal trauma history in the mental health professionals, 13 of the studies used percentages to present their data, with prevalence ranging from 21.2% (Somoray et al., 2017) to 83.3% (Corbett‐Hone & Johnson, 2022). Taking the three main study locations used, we can compare the proportion of MHPs who have experienced trauma with those within general populations (Table 4). This suggests that in American, Australia and the UK, significantly higher proportions of MHPs experienced trauma compared with the general population of their own country.

**TABLE 4:** Comparison of mental health professionals personal trauma history with general populations.

Location	National figure	Mental health professionals
USA	5% (National Centre for PTSD, 2022)	Range from 38.7% (Adams & Riggs, 2008) to 71.2% (Chaverri et al., 2018)
Australia	5%–10% (Phoenix Australia, 2022)	Range from 21.2% (Somoray et al., 2017) to 37.1% (Dunkley & Whelan., 2006)
UK	3% (Patient UK PTSD, 2022).	59.3% (Sodeke‐Gregson et al., 2013)

Abbreviations: PTSD, post‐traumatic stress disorder; USA, United States America; UK, United Kingdom.

## 5 DISCUSSION

The key findings of our review are as follows: firstly, that personal trauma history and secondary traumatic stress are common in mental health professionals; and secondly, that we identified an association between a mental health professional's personal trauma history and secondary traumatic stress symptoms. The prevalence of a personal trauma history ranged from 19% to 83.1%, with mental health professional's experiencing personal trauma history in 22 of the 23 studies captured in our review. This means that those professionals who work in the field of mental health are likely to have experienced their own trauma. Eighteen studies reported on whether mental health professionals' experienced secondary traumatic stress, and prevalence ranged from 19.2% to 70%. This signifies that mental health professionals who work with people who have been traumatized are at risk of experiencing secondary traumatic stress.

Our results found that 13 of the 18 studies which investigated the association between personal trauma history and secondary traumatic stress found a statistically significant positive relationship between these variables, albeit the correlation is of small magnitude. This means that mental health professionals with a personal history of trauma are at heightened risk of suffering from secondary traumatic stress when working with those who have been traumatized. Our findings on the correlation between personal trauma history and secondary traumatic stress are in keeping with those reported in the review by Leung et al. (2022), which adopted a broader definition of mental health professionals.

Our findings should be interpreted with some caution due to methodological weaknesses of the included studies. All the studies captured in this review were cross‐sectional in design. Several authors identified this as a limitation of their research and recommended that future longitudinal research take place to assess for causation (Creamer & Liddle, 2005; La Mott & Martin, 2019; Linley & Joseph, 2007; Makadia et al., 2017; Ray et al., 2013; Somoray et al., 2017). Furthermore, using a yes/no question to determine the presence of past trauma does not take into consideration that trauma can be viewed differently, and perceptions can vary (Dunkley & Whelan, 2006). Asking the participant to respond using either a yes or no does not consider the characteristics of trauma. For example, a history of interpersonal trauma (child abuse) versus impersonal trauma (natural disaster). It also fails to measure the impact of the trauma or the influence of previous therapy. A valid assessment tool is needed to measure for personal trauma and anticipate for these factors. Our estimates of the prevalence of personal trauma and secondary traumatic stress will also be influenced by selection bias, in that participants with personal experience of these issues may have been more likely to participate in studies compared to mental health professionals with no experience of such issues.

The definition of trauma is understood quite broadly by participants, and it may be that those recruited into the studies did not, in fact, work with trauma but other psychological difficulties and yet identify themselves as trauma therapists (Makadia et al., 2017; Pearlman & Mac Ian, 1995). Finally, some studies had a low response rate which limited the generalisability of their findings (Adams & Riggs, 2008; Creamer & Liddle, 2005; Devilly et al., 2009; Killian, 2008; Mangoulia et al., 2015).

### 5.1 Implications for training and practice

There is a common acknowledgment in the workplace that those attracted to a career in this field of mental health are often those with some knowledge and experience of difficult life events. Despite this, there is lack of acknowledgment within mental health professional's training to help prepare the student for when they are in practice. Embedding teaching about the possible psychological impacts of secondary traumatic stress for those mental health professionals with their own experiences of trauma could lead to improved well‐being of the practitioner and support the services to retain skilled staff. It would mean that, when in practice those that are able to identify themselves at increased risk of suffering from secondary traumatic stress, could receive targeted support from the organization with the development of specialized supervision and debriefs. As found in Dunkley & Whelan's. (2006) study where strong supervision reduced disruptions in clinicians' belief symptoms.

### 5.2 Implications for research

Further research would benefit from adequately‐powered studies with a prospective design, to understand the association of personal trauma history and secondary traumatic stress over time. Studies should use validated tools to measure personal trauma history and secondary traumatic stress, and the field would benefit from consensus about which tools have the best psychometric properties, which would require a review of the evidence. In terms of prospective designs, it would be important to investigate whether there are particular periods of time when mental health professionals with a history of personal trauma history are more vulnerable to developing secondary traumatic stress, such as soon after qualification. Qualitative studies are also needed: firstly, to develop a richer understanding of the mechanisms by which a personal trauma history might be associated with secondary traumatic stress, as well as relevant risk and protective factors; secondly, to inform hypotheses for future research; and thirdly, to better understand what services, supervisors and trainers can put in place to help and assess the impact of this.

### 5.3 Limitations

Publications included were written in English, meaning that we may have missed studies, particularly from Global Majority countries. Ethnicity was also not discussed in many of the studies captured in the review and therefore places limits on the generalisability of the findings. Given that studies typically report small correlations between personal trauma history and secondary traumatic stress, many of the studies in our review are likely to be underpowered. Studies reporting on the prevalence of personal trauma and secondary traumatic stress may have also been influenced by selection bias where those experiencing these issues would be more likely to participate in their studies. Another limitation of this review was that all the papers captured were of cross‐sectional design. This would have excluded those mental health professionals who may have left the workplace after developing secondary traumatic stress, as well as limiting our understanding about the relationship between personal trauma history and secondary traumatic stress over time. For example, it is not clear at what point in their careers mental health professionals are most likely to develop secondary traumatic stress, which makes it harder for educators, supervisors and managers to know who is most vulnerable in terms of career stage. It could also be argued that trainees have not qualified as mental health professionals and should not have possibly been included in the review. The prevalence of personal trauma history and secondary traumatic stress ranged widely across studies, which likely reflects differences in how these variables were measured, as well as possible variation across different mental health professional groups.

## 6 CONCLUSION

In conclusion, our review highlights that both personal trauma history and secondary traumatic stress are common in mental health professionals. Across our included studies, we found that personal trauma history and secondary traumatic stress were present in at least one fifth of mental health professionals, whilst at the higher end of the range, personal trauma history was present in over four fifths of mental health professionals, whilst secondary traumatic stress was present in over two thirds of mental health professionals. Secondly, we found that personal trauma history is associated with secondary traumatic stress, suggesting that personal trauma history is a potential risk factor for developing secondary traumatic stress. This means that those involved in training, supervising and managing mental health professionals should be aware that a high proportion of the mental health workforce has a history of trauma, and that this history may put such professionals at higher risk of developing traumatic stress. This is likely to have major consequences for staff wellbeing and retention. More research is needed to understand the relationship between personal trauma history and secondary traumatic stress in greater depth, for instance through qualitative studies. Quantitative studies adopting prospective designs are also a priority, which can provide insights into both risk and protective factors for those mental health professionals with a personal trauma history. Improving the evidence base is required so that mental health professionals with a history of personal trauma can potentially be at reduced risk of developing secondary traumatic stress.

## 7 RELEVANCE TO MENTAL HEALTH NURSING

This systematic review identified that mental health professionals with a personal history of trauma are at heightened risk of suffering from secondary traumatic stress. In specialized areas like mental health, retention of professionals, including nurses is a fundamental issue. This study identifies those mental health professionals who are at higher risk of developing secondary traumatic stress, enabling health care providers to ensure additional and targeted support is given.

## AUTHOR CONTRIBUTIONS

All authors listed met the authorship criteria according to the latest guidelines of the International Committee of Medical Journal Editors, and that all authors are in agreement with the manuscript.

## CONFLICT OF INTEREST STATEMENT

There is no conflict of interest.

## Supporting information


Data S1.

## Data Availability

Data sharing not applicable to this article as no datasets were generated or analysed during the current study.
