# Supplementary material for: Personal trauma history and secondary traumatic stress in mental health professionals: A systematic review
Source: J Psychiatr Ment Health Nurs. 2024 Jul 7;32(1):13–30. doi: 10.1111/jpm.13082 (PMC11704991; doi:10.1111/jpm.13082)
Supplement: Supplementary file 1 — Data S1. [file JPM-32-13-s001.docx]

**SUPPORTING DOCUMENTS**

**DOCUMENT 1: Evaluation of the differences between Leung et al., (2022) published review and the authors.**

|  | Leung et al’s work | Author’s review |
| --- | --- | --- |
| Search strategy | | |
| Search term | “Burnout” or “secondary traumatic stress” or “compassion fatigue” or “critical incident stress” or “indirect trauma” or “PTSD” or “traumatic stress” OR “indirect trauma” AND “professional” or “worker” or “provider” or “volunteer*” or “clinician*” or “counsellor*” or “therapist” * or “trainee*” or “student*” or “interviewer” or “advocate” or “interviewer” or “child protection” or “child welfare” or “victim services or “protective services”. | Search terms were identified with a librarian and tested using a scoping review.  Keywords “Seconda* trauma” or “secondary traumatic stress” or “compassion fatigue” or “vicarious trauma*” and no date limit.  Burnout was not used as a search term as this is caused by organisational pressure not by working with those who have been traumatised.    An intentionally broad search was used for personal trauma because of the nonspecific use of outcome measures across the various terms.  Mental Health Professionals was not used as a search due to the wide variation of the term used in practice. This was screened using a robust inclusion and exclusion criteria and reviewers’ expertise from working in this field.  Those working within protective and victim services, child welfare, volunteers, providers and interviewers cannot be guaranteed to be MHPs. Also Leung’s study includes student and trainee but does not state which profession and did not include mental health nurses which is the majority of the mental health professional population. |
| Databases | “Interdisciplinary databases ie Cochrane library, JSTOR, PsychInfo and Pubmed”. The review was limited to the first 1000 results captured. | EMBASE, PsyInfo, Web of Science and CINHAL.  There was no limitation on the numbers captured and references of papers were hand searched including relevant systematic reviews for additional studies meeting the criteria.  In addition, google scholar was searched using the same keywords, and published articles on STS, VT and CF were surveyed with the aim of identifying any appropriate articles not found in the database search. |
| Study selection | Articles were selected and screened by a single reviewer. A second reviewer was only used if it was difficult to determine if an article met the inclusion criteria. | Two authors (AH & XH) independently searched for the journals using the search terms and identified the same articles.  All articles were screened independently by the two reviewers (AH & XH) using the inclusion and exclusion criteria. Discussions were held between the two reviewers if needed and referred to a third independent reviewer (TJ) if necessary. |
| Time | Peer reviewed papers published from 2000 to June 2021 | To ensure a full and robust review of a concept with no rationale to limit a time frame there was no time limit. All initial searchers were run in April 2022, and then final search was re-run in August 2023. |
| Screening criteria | | |
| Inclusion criteria | Peer reviewed journals.  Which discussed BO, STS or VT as an outcome of variable interest. It did not include CF despite being recognised widely in the field for its STS symptoms and confusingly they have included this as a search term. There is no mention of a valid tool being used to assess BO,STS or VT.  If the study included or measured Personal trauma.  Those working within protective and victim services, child welfare, volunteers, providers and interviewers cannot be guaranteed to be MHPs. Also, Leung’s study includes student and trainee but does not state which profession and did not include mental health nurses which is the majority of the mental health professional population  Used both quantitative and qualitative data to examine the association between personal trauma and BO, STS or VT.  Written in English.  Published from 2000 to June 2021 with no rationale why 2000 was chosen. | Peer reviewed journal  Valid measure used to capture STS, CF or VT.  Mental Health Professionals working in a mental health setting to ensure exposure to those experiencing trauma.  Reported on the prevalence of either personal trauma history and STS/VT and CF, as well as reported on either the correlation between personal trauma history and STS/CF or VT.  Used expertise to determine whether the sample group were a mental health professional. This included the following mental health nurses, psychiatrists, psychotherapists, therapists, social workers, psychologists, occupational therapists, and counsellors.  Written in English  No limitations of publication to capture the full range of data available to fully explore this possible association. Searches took place up until August 2022. |

**DOCUMENT 2**

**PsychINFO**

**Date: No limit**

| **Search Term** | | **Fields** | **Exact Query** |
| --- | --- | --- | --- |
| 1 | Seconda* trauma | Title & Abstract | Seconda* trauma.tw |
| 2 | Secondary traumatic stress | Title & Abstract | Secondary traumatic stress.tw. |
| 3 | Compassion Fatigue | Title & Abstract | Compassion fatigue.tw. |
| 4 | Compassion Fatigue | Title & Abstract | Exp Compassion Fatigue |
| 5 | Vicarious trauma* | Title & Abstract | Vicarious.trauma.tw |
| 6 | 1 OR 2 OR 3 OR 4  OR 5 | Title & Abstract | 1 OR 2 OR 3 OR 4 OR 5 |
| 7 | Limit search | Title & Abstract | Quantitative, Peer journal, English, Doc type: journals, human |

**DOCUMENT 3: Adapted Newcastle-Ottawa tool for risk of bias**

| **Criteria** | **Maximum Score** |
| --- | --- |
| *Cross-sectional Studies* | |
| Sample representative of target sample (e.g. all eligible or random sample)?   - Score 2 if truly representative of the average in the target population; all subjects, or random sampling employed and response rate >50%. - Score 1 if somewhat representative of the average in the target group. Uses non-random sampling (selected group of users/convenience sample) or random sampling with response rate <50%. - Scores 0 if no description of the derivation of the included subjects. | 2 |
| Sample size justified and satisfactory? | 1 |
| Non-response rate defined, satisfactory, and characteristics of responders/non-responders compared? | 1 |
| Ascertainment of exposure (i.e., personal trauma history) valid and/or well-described? | 1 |
| Assessment of outcome with a robust tool and/or record linkage? (i.e., validated tool such as ProQOL, STSS, IES-R, TABS-5, STES, PCL, TSIBS)   - Score 2 if used one of the listed measures - Score 1 if used a different measure - Score 0 for non-validated measures | 2 |
| Outcome per group reported appropriately? (statistical test) | 1 |
